# Using 2-deoxy-2-[^18^F]fluoro-D-glucose ([^18^F]FDG) to study carbon allocation in plants after herbivore attack

**DOI:** 10.1186/s13104-015-0989-z

**Published:** 2015-02-18

**Authors:** Stefan Meldau, Melkamu G Woldemariam, Amol Fatangare, Ales Svatos, Ivan Galis

**Affiliations:** Department of Molecular Ecology, Max-Planck-Institute for Chemical Ecology, Hans-Knöll-Str.8, 07745 Jena, Germany; German Centre for integrative Biodiversity Research (iDiv), Deutscher Platz 5, 04107 Leipzig, Germany; Present address: KWS SAAT AG, Molecular Physiology, R&D, RD-ME-MP, Grimsehlstrasse 31, D-37555 Einbeck, Germany; Present address: Boyce Thompson Institute for Plant Research, 533 Tower Road, Ithaca, 14853 NY USA; Mass Spectrometry Research Group, Max-Planck-Institute for Chemical Ecology, Hans-Knöll-Str.8, 07745 Jena, Germany; Present address: Okayama University, Institute of Plant Science and Resources, Chuo 2-20-1, 710-0046 Kurashiki, Japan

**Keywords:** 2-deoxy-2-[^18^F]fluoro-D-glucose ([^18^F]FDG), Herbivory, Jasmonate signalling, *Nicotiana attenuata*, Fatty acid-amino acid conjugates

## Abstract

**Background:**

Although leaf herbivory-induced changes in allocation of recently assimilated carbon between the shoot and below-ground tissues have been described in several species, it is still unclear which part of the root system is affected by resource allocation changes and which signalling pathways are involved. We investigated carbon partitioning in root tissues following wounding and simulated leaf herbivory in young *Nicotiana attenuata* plants.

**Results:**

Using 2-deoxy-2-[^18^F]fluoro-D-glucose ([^18^F]FDG), which was incorporated into disaccharides *in planta*, we found that simulated herbivory reduced carbon partitioning specifically to the root tips in wild type plants. In jasmonate (JA) signalling-deficient *COI1* plants, the wound-induced allocation of [^18^F]FDG to the roots was decreased, while more [^18^F]FDG was transported to young leaves, demonstrating an important role of the JA pathway in regulating the wound-induced carbon partitioning between shoots and roots.

**Conclusions:**

Our data highlight the use of [^18^F]FDG to study stress-induced carbon allocation responses in plants and indicate an important role of the JA pathway in regulating wound-induced shoot to root signalling.

## Background

Plants face a dilemma when stressed by wounding or herbivore attack - to invest resources into defence reactions or into growth processes. Research on how plants solve this dilemma is important for understanding the evolution of resistance and tolerance strategies of plants, and helps to facilitate the development of crop improvement strategies. The production of defensive metabolites is tightly linked to the wound- and herbivory-induced activation of defence hormones, including jasmonic acid (JA) and its isoleucine conjugate JA-Ile [1]. Activation of JA-dependent resistance pathways is often accompanied by changes in the levels of primary metabolites, such as sugars, amino acids and organic acids, which serve as substrates and precursors or provide energy required for defence metabolite biosynthesis [2-7]. Although the wound- and herbivory-induced signalling or treatment with JA increase a plant’s response to herbivore attack [8], activation of the JA pathway can limit the availability of resources required for plant growth and fitness [9-12].

Biotic and abiotic stress can increase sink strength of certain plant tissues; a common response in many plant species, including carrot [13], tomato [14], hybrid poplar trees [2,15,16] and pea [17]. However, the opposite response also occurs, such as the flow of carbon away from stressed tissues, often to storage organs, such as roots [7,18-20]. But the direction of resource re-allocation can change with environmental conditions and plant ontogeny. For example, in *Arabidopsis thaliana*, 2-deoxy-2-[^18^F]fluoro-D-glucose ([^18^F]FDG), a radioactive tracer that is used to study carbohydrate allocation, is transported mainly to the root system in vegetative plants but is directed to above-ground tissues when plants enter the reproductive stage [21].

One of the best plant model systems to study responses upon herbivore attack is *Nicotiana attenuata*, an annual plant that grows in the post-fire environment in the Great Basin Desert (Utah, USA). The interaction between *N. attenuata* and its Lepidopteran herbivore *Manduca sexta* has been intensively studied. During *M. sexta* attack, fatty acid-amino acid conjugates (FACs) present in the herbivores’ oral secretions (OS) are rapidly recognized by *N. attenuata*; FACs amplify and modify wound-induced responses in *N. attenuata*, including the biosynthesis of JA and JA-Ile [22,23]. The biosynthesis of JA-Ile and its consequent perception through SCF^COI1^ results in transcriptional reprogramming that leads to the accumulation of various anti-herbivore secondary metabolites [10,24-26]. JA-mediated herbivory-induced responses are associated with large fitness costs in *N. attenuata* [11], demonstrating the trade-off between plant growth and defence. However, it is not known whether, in *N. attenuata*, the JA pathway orchestrates the resource allocation changes that follow herbivore attack.

Schwachtje and colleagues found that simulated herbivory increases partitioning of recently assimilated carbon to roots of *N. attenuata* plants; a response that has been linked to a process termed as “herbivory-induced resource sequestration” [7,19,20,27-31]. The role of the extra carbon in the below-ground parts remains unknown: it could be utilized for growth of the roots, be stored within the root system, or help in the synthesis of defence compounds such as nicotine. However, it was shown recently that herbivory reduces sugar levels and starch in the roots of *N. attenuata* [32]. This depletion of carbon resources correlates well with reduced growth of the primary root after wounding and simulated herbivory [33,34] and with a diminished ability to regrow and tolerate herbivore attack [32]. Until now, it has been unclear in which parts of the *N. attenuata* root system these changes in carbon allocation are manifested.

We used the short-lived isotope ^18^F in simulated herbivory experiments with leaf-application of the sugar analogue [^18^F]FDG to analyse carbon allocation at a fine spatial scale in the root system. In addition, we analysed the role of the JA pathway in herbivore-induced [^18^F]FDG distribution by using transgenic plants silenced in the expression of COI1. Our results demonstrate that [^18^F]FDG partitioning to root tips is strongly reduced after leaf herbivory. Plants silenced in COI1 expression reveal a distinct role of JA perception in [^18^F]FDG distribution after wounding.

## Methods

### Plant cultivation

Transgenic irCOI1 *N. attenuata* plants were described elsewhere [25]. These lines are transformed with inverted-repeat constructs, allowing reduced transcript levels of the gene involved in JA perception (irCOI1). For [^18^F]FDG experiments, cultivation of *N. attenuata* plants was described elsewhere [35], with the following modifications: 14 d old seedlings were transferred from Petri dish to sand (0.7-1.2 mm grain size, Raiffeisen GmbH, Germany) and fertilized with 0.15 gL^−1^ Ferty B1 (Planta Düngemittel GmbH, Regenstauf, Germany); 0.25 gL^−1^ Ca_2_(NO_3_). A small lid was placed over the plants to avoid drought stress. After three days, the lid was moved to allow some air exchange, and after five more days the lid was removed completely. Twelve days later, the plants were transferred to hydroponic solution (for 1 L: 0.1929 g Ca_2_SO_4_; 0.1232 g Mg_2_SO_4_, 0.0479 g K_2_HPO_4_, 0.0306 g KH_2_PO_4_ and 0.5 mL micronutrient solution (for 1 L: 2.533 g H_3_BO_3_; 1.634 g MnSO_4_, 0.151 g Na_2_MoO_4_, 0.08 g CuSO_4_, 0.02 g CoCl_2_, 0.5 mL Fe-DTPA (for 1 L: 2.78 g FeSO_4_, 3.93 g Titriplex (Merck KGaA, Darmstadt, Germany))). Plants were grown in growth chambers under 16 h light (133 μmol m^−2^ s^−1^) at 22°C and 65% humidity.

### TLC plate analysis

We used one WOS-treated plant to analyze if [^18^F]FDG can be metabolized by *N. attenuata* plants. We applied 5 μL of [^18^F]FDG to a single punctured wound of a source-sink transition leaf of a 4.5 weeks old WT plant. Another younger leaf was treated with WOS. After 8 h, the plant was disassembled and leaf and root tissues (50 mg) were extracted with MeOH. 15 μL of the extract was applied to a 0.2 mm HPTLC silica gel 60 F254 plate (Merck) and chromatography was done using acetonitrile–water (17:3, v/v), containing 0.05% of 2-aminoethyl diphenylborinate. After chromatography, the plate was sprayed with detection reagent (4 g of diphenylamine and 4 mL of aniline dissolved in 160 mL of acetone, 20 mL of conc. H3PO4 added and filled to 200 mL with acetone) and heated up to 120°C for two minutes until bands were clearly visible. The plate was then transferred to an imaging cassette, covered with a positron imaging plate and scanned after 1 h exposure (FLA 3000 system, Fujifilm, Tokyo, Japan).

### [^19^F]FDG experiments

Three mature rosette leaves from each plant were selected for stable-isotope-labelled [^19^ F]FDG application. Leaves were wounded on leaf lamina on either side of the midrib using micropipette tip. Five μL of [^19^F]FDG (20 mg mL^−1^, Sigma Aldrich, St. Louis, MO, USA) solution was immediately applied on each wounded region. After 30 min, 5 μL of water was applied on the same region to aid [^19^F]FDG uptake. Four hours after treatments, the leaves were harvested and extracted using slightly modified methanol/chloroform extraction procedure [36]. In brief, leaves were ground in liquid nitrogen. Methanol (1.5 mL) containing ^13^C labeled glucose (10 μg mL^−1^, Sigma Aldrich, St. Louis, MO, USA) and chloroform (0.75 mL) were added to the tissue sample. The mixture was sonicated in ultrasonic bath (Merck, Eurolab NV, Belgium) for 15 min at room temperature. After sonication, water (0.5 mL) and chloroform (0.5 mL) was added to the sample. Sample was centrifuged at 4000 g for 15 min at 4°C. Supernatant was concentrated using the rotating vacuum dryer (Concentrator 5301, Eppendorf Vertrieb, Germany). Dried supernatant sample was resuspended in 0.1 mL of water and stored at −80°C until further LC-MS analysis.

### LCMS and LCMS^n^ measurements

LC-MS data were acquired using Dionex UltiMate 3000 UHPLC system coupled to Thermo-Fisher LTQ-Orbitrap XL hybrid mass spectrometer (both Thermo Fisher Scientific, Bremen, Germany). The extracts were separated on Supelco apHera NH2 column (15 cm × 4.6 mm, particle size- 5 μm) at room temperature. The mobile phase consisted of water (A) and acetonitrile (B). Elution gradient was set as follows: 20% A (0 min), 20% A (0.5 min), 45% A (13 min), 45% A (18 min) and 20% A (20 min). The mobile phase flow rate was 1 mL min^−1^ and the injected volume was set at 2 μL. Electrospray ionization (ESI) source was used for ionization of LC eluate in negative ion mode. Capillary temperature was 280°C, and sheath and auxiliary gas flow rates were 50 and 10 arb (arbitrary units), respectively. The sweep gas flow rate was set at 5 arb and source voltage at 4 kV. The capillary voltage and tube lens were set at −47 V and −120 V, respectively. During LCMS measurements, FTMS resolution was set to 100,000 and samples were analyzed in full scan mass range of m/z 100–800 with the acquisition of profile-type mass spectra. During LCMS^n^ measurements, LC peak retention time (RT) window was given to acquire MS/MS spectra of few selected ions in that RT window. All other parameters were identical to that of LCMS. MS/MS spectrums were acquired at a FT resolution of 15,000 at collision energies of 5, 10, 20 and 30 respectively and with isolation window of 1.6 Da. The raw data was processed and compared using Xcalibur version 2.0.7 (Thermo Fisher Scientific, Bremen, Germany). The mass accuracy error threshold was fixed at 5 ppm.

### [^18^F]FDG experiments

**Figure 1:**
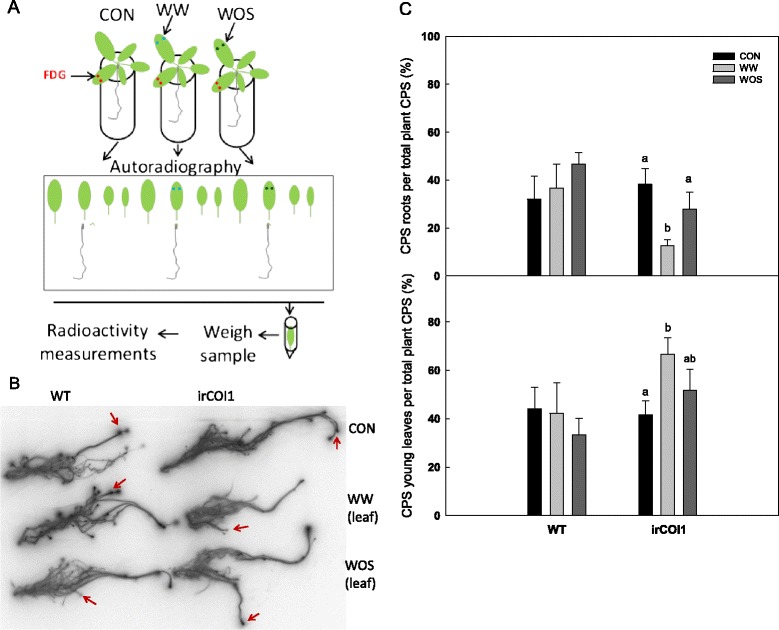
**Radioactivity accumulation after [** ^**18**^ **F]FDG labelling and simulated herbivory in** ***N. attenuata.*** **(A)** Scheme of the experimental setup. Leaves of 3 week old plants were punctured with a needle and applied with FDG (red dots). Another leaf kept untreated (CON) or was wounded and treated with 1 μL of water (WW, blue dots) or 1 μL of 1:5 diluted *Manduca sexta* oral secretions (WOS, green dots). After 8 h the plants were disassembled and an autoradiographic picture was taken from a set of plants. Plant parts were then weighed and radioactivity was measured. **(B)** Autoradiographs of roots. Red arrows indicate changes at root tips. **(C)** Radioactivity measurements in roots and sink leaves of [^18^F]FDG-labelled wild type (WT) and inverted repeat COI1 (irCOI1) plants. Letters indicate significant differences between treatments (ANOVA, root: F2,12 = 5.16; P = 0.077; young leaves: F2,12 = 3.06; P = 0.0301), N ≥ 5 ± SE.

Using size-matched, early rosette-stage plants (rosettes of approximately 5 cm radius), 1 μL of [^18^F]FDG solution (1.5-2 MBq μL^−1^; in H_**2**_O, FCON, Holzhausen a.d. Haide, Germany) was applied to puncture wounds made on both sides of the midrib of the third oldest leaf (Figure 1A). Four hours after the application of [^18^F]FDG, 5 μL of water was applied to the wounds to aid uptake of remaining FDG on the leaf surface. Treatments were applied immediately after tracer application (see Figure 1A) to the leaf next younger to the load leaf. This leaf was either left untreated (CON), or was puncture-wounded in two places, with application of 1 μL water (WW) or 1:5 diluted *M. sexta* oral secretions (WOS). For FAC treatments, leaves of three week old plants were punctured with a needle and applied with [^18^F]FDG. Another leaf was wounded and treated with 1 μL of water (WW) or 1 μL of the fatty acid-amino acid conjugate N-linolenoyl-glutamate (WFAC), at a concentration similar to *M. sexta* OS [37]. Eight hours after these treatments, all leaves, shoot-root junction and roots were carefully separated, transferred to an imaging cassette, covered with a positron imaging plate and scanned after 1 h exposure (FLA 3000 system, Fujifilm, Tokyo, Japan). For radioactivity measurements, plant parts were transferred to plastic tubes and radioactivity was measured with a well counter (Isomed 2100, Nuklear Medizintechnik Dresden GmbH, Dresden, Germany).

## Results and discussion

**Figure 2:**
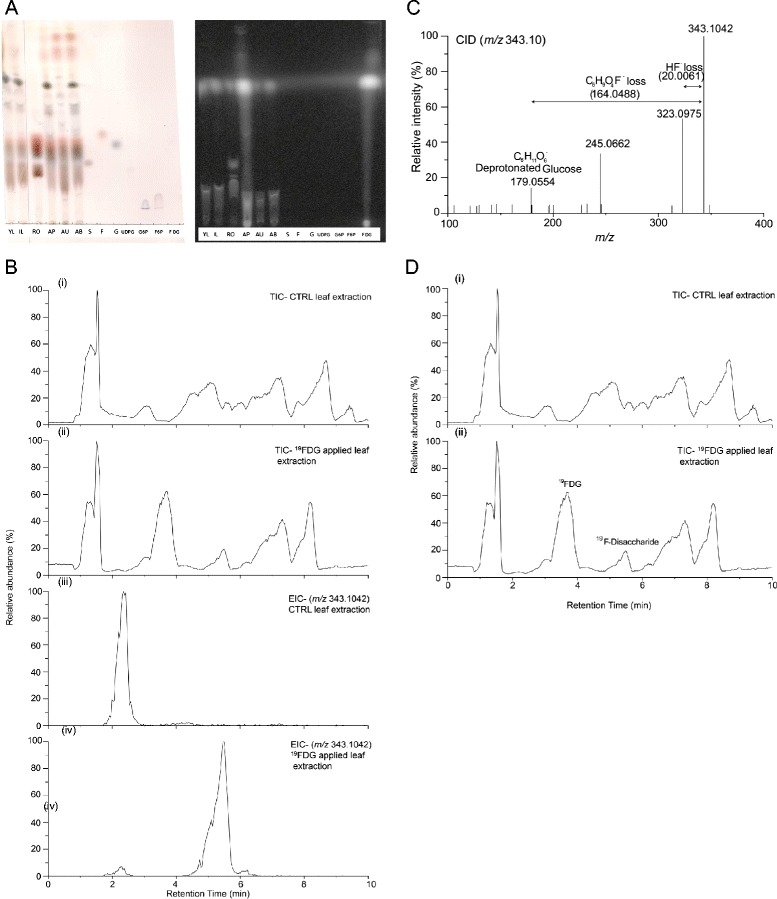
**[** ^**18**^ **F]FDG and [** ^**19**^ **F]FDG metabolism in** ***N. attenuata*** **leaves. (A)** [^18^F]FDG is metabolized in *Nicotiana attenuata*. One leaf (AP) of a 3.5 week old plant was punctured with a needle and applied with 5 μL [^18^F]FDG solution. Another leaf was induced with wounding and treated with 1 μL of 1:5 diluted *Manduca sexta* oral secretions (IL). After 8 hours the plants were disassembled, tissues were extracted and qualitative sugar analysis was done by performing thin layer chromatography (TLC, left picture). Autoradiograph was taken of the same TLC plate (right picture). Labeling: YL = youngest leaves, IL = induced leaf, RO = root, AP = apical part of the [^18^F]FDG treated leaf, AU = apical bud of the plant, AB = basal part of the [^18^F]FDG treated leaf, standards: S = sucrose, F = fructose, G = glucose, UDPG = uridindiphosphate-glucose, G6P = glucose-6-phosphate, F6P = fructose-6-phosphate, FDG = [^18^F]FDG. **(B)** Comparison of total (TIC) and extracted ion chromatograms ([^19^ F]FDG disaccharide: m/z 343.10) of leaf extract (ctrl, i and iii) and [^19^F]FDG applied leaf extract (ii and iv). **(C)** MS^2^ of m/z: 343.10 (retention time: 5.50 min). **(D)** Comparison TIC of CTRL-leaf extract (i) with [^19^F]FDG applied leaf extract (ii) for depicting [^19^F]FDG and [^19^F]-disaccharide chromatographic peaks.

**Figure 3:**
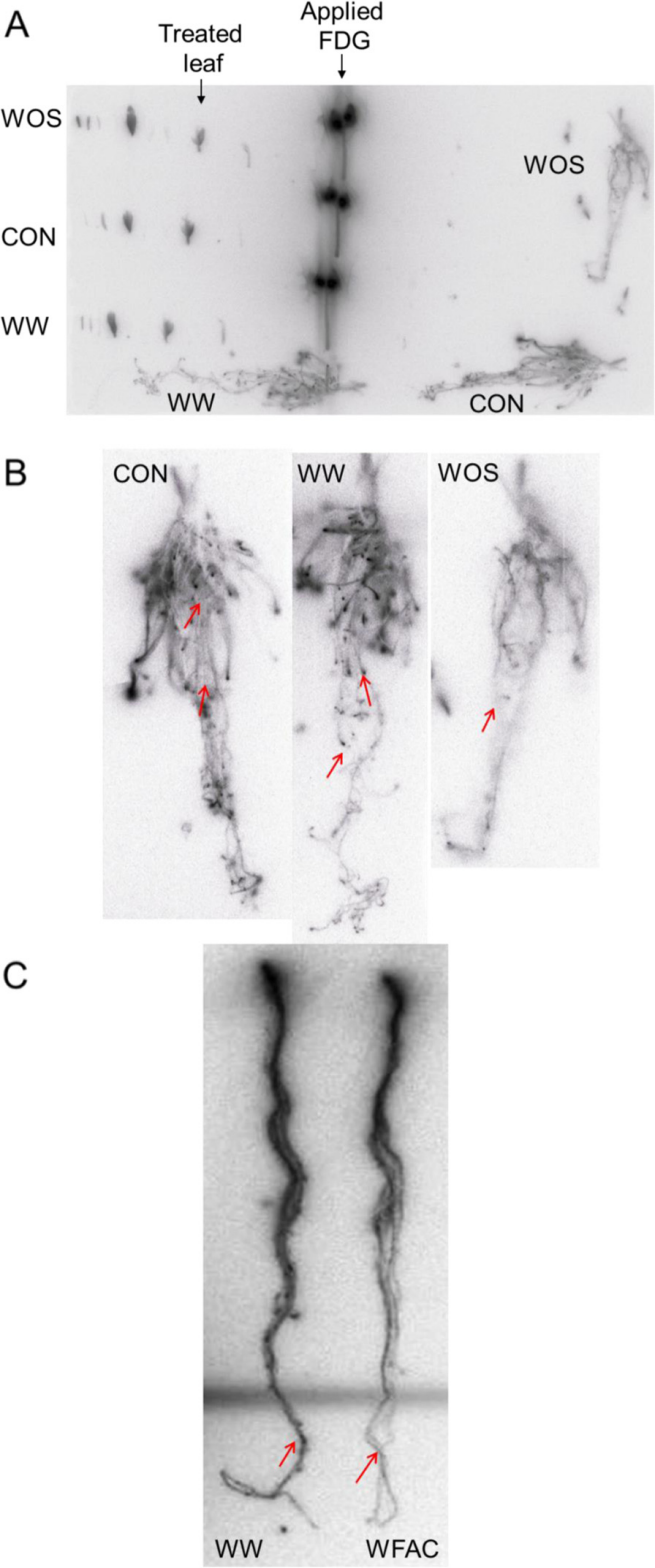
**[** ^**18**^ **F]FDG distribution after simulated herbivory in** ***Nicotiana attenuata*** **plants. (A)** Autoradiograph from plant parts of [^18^F]FDG-treated wild type *N. attenuata* plants. Leaves of 3.5 week old plants were punctured with a needle and applied with [^18^F]FDG. Another leaf kept untreated (CON) or was wounded and treated with 1 μL of water (WW) or 1 μL of 1:5 diluted *Manduca sexta* oral secretions (WOS). After 8 hours the plants were disassembled and an autoradiographic picture was taken. **(B)** Root pictures from **(A)** were magnified and assembled next to each other to demonstrate the reduced accumulations of radioactivity after WOS treatments. **(C)** Autoradiographed roots of [^18^F]FDG-treated *N. attenuata* plants. Leaves of three week old plants were punctured with a needle and applied with [^18^F]FDG. Another leaf was wounded and treated with 1 μL of water (WW) or 1 μL of the fatty acid-amino acid conjugate N-linolenoyl-glutamate (WFAC), one of the active elicitors in *M. sexta* oral secretions.

It has been suggested that [^18^F]FDG, a radioactive glucose analogue, could be used as a tracer for photoassimilates distribution in plant studies [38]. Although, [^18^F]FDG uptake and metabolism has been extensively studied in animal cells [39-41], its metabolism in plant tissues is not well characterized. First, we performed thin layer chromatography (TLC) experiments to analyze whether [^18^F]FDG is metabolized in *N. attenuata* plants, as has been shown in *A. thaliana* [21]. The detection of multiple radioactive bands in local and systemic leaf and root tissues suggest that [^18^F]FDG is taken up, transported and metabolized by the plant (Figure 2). To further characterize the metabolism of FDG in plants, we supplied the stable-isotope-labelled [^19^F]FDG to plant leaves and analysed [^19^F]FDG metabolites via liquid chromatography-mass spectrometry (LC-MS). In all extracts from [^19^F]FDG-labelled leaves, we found a peak eluting at retention time of 5.4 min with m/z 343.1042 and with calculated monoisotopic mass of C_12_H_20_O_10_^19^F^−^ (±4 ppm, Figure 3). Upon fragmentation, m/z 343.1042 gave rise to secondary ions m/z 323.0975 and 179.0554. The first fragment can be rationalized by neutral loss of HF (20.0061), whereas the other fragment ion was identified as deprotonated glucose (C_6_H_11_O_6_^−^). Retention time of the new compound was found to be between [^19^F]FDG and sucrose retention times. Taken together, our data show the *in planta* incorporation of [^19^F]FDG into different metabolites, including disaccharides, presumably [^19^F] sucrose.

Since FDG is a metabolically active compound in *N. attenuata*, we measured effects of simulated herbivory treatments on the distribution of the radioactivity after exogenous administration of [^18^F]FDG. When we analysed the distribution of ^18^F in wild-type plants, root tips of control and WW-treated plants accumulated high concentrations of ^18^F-radioactivity relative to the root axes; however, the accumulation of ^18^F in root tips was highly reduced after simulated herbivory (WOS) (Figure 3A, B; Figure 1B). The highly localized reduction of recently assimilated carbon after WOS treatments in root tips were also found in experiments with radiolabeled CO_2_ (Lilian Schmidt and Michael Thorpe, personal communication). There was also a reduction in ^18^F at root tips after leaves were treated with FACs, the active elicitors in the oral secretions of *M. sexta* (Figure 3C). In above-ground tissues, radioactivity accumulated mostly in young leaves and in the shoot-root junction (data not shown), but there were no apparent effects of WW or WOS.

Because root responses after simulated herbivory, such as sugar levels, root growth inhibition and plant re-growth, has been shown in *N. attenuata* to partially depend on JA-perception through NaCOI1 [32,34], we tested the hypothesis that the distribution of [^18^F]FDG or its metabolites depend on JA-signalling. In addition to imaging tracer distribution, we also quantified tissue radioactivity by gamma counting in this experiment (see Figure 1A for experimental outline). In contrast to the strong WOS-treatment effect apparent in the autoradiographs for ^18^F-accumulation in root tips, ^18^F-content of the entire root system showed no significant differences (nor did leaf tissues, Figure 1C). Apparently, the treatments induced a highly localized response at root tips, which was not detectable when the entire root system was analysed. In plants silenced in NaCOI1 expression (irNaCOI1, [25]), autoradiography showed that the fraction of ^18^F in their root tips was markedly reduced after WW treatment and also, to some extent, after WOS (Figure 1B). Further, the radioactivity distribution (Figure 1C) showed a significant effect of the WW treatment, and not for WOS. After WW, NaCOI1 plants showed a change in distribution in favour of the young leaves, at the expense of the roots. These responses contrast with those in WT plants, where none of the treatments significantly affected whole organ ^18^F distribution. Taken together, these data demonstrate that simulated herbivory altered the accumulation of [^18^F]FDG or its metabolites specifically in root tips, and that JA perception is important for resource allocations to roots of wounded plants. The higher accumulation of radioactivity in younger leaves in COI1-silenced plants indicates that JA signalling alters carbon allocation between shoots and roots. Clearly more research is needed to identify the mechanisms behind the effects of JA on root responses after leaf wounding.

Two reports in *N. attenuata* show that leaf herbivory specifically induces changes in carbon allocation to roots [7,32]. While Schwachtje et al. [7] found that simulated herbivory increases allocation of recently assimilated ^11^CO_2_ to roots, they did not find increases in root carbohydrate pools. In contrast, Machado et al. [32] recently demonstrated that leaf herbivory in *N. attenuata* reduced root carbohydrate pools and negatively influenced plant tolerance responses measured as plant re-growth [32]. In addition, while JA signalling did not affect carbon allocation to roots in the Schwachtje et al. [7] study, Machado and colleagues found that sugar and starch levels did not change in COI1-silenced plants. Our results support the notion that *N. attenuata* does not “bunker” carbon resources in root after leaves are attacked but rather that allocation within the root is altered. In vegetative *A. thaliana* plants, wounding and MeJA application to leaves did not result in increased allocation of [^18^F]FDG or its metabolites to the root system [21], which suggests that different plant species at similar ontogenic stages may not only have different responses of root growth [42], but also have different resource allocation strategies when responding to herbivory. In agreement with this, Diezel and colleagues reported a strong effect of ontogeny on the response of *N. attenuata* plants to herbivory [43]. Our results may differ from those of Schwachtje et al. [7] because their plants were at a late-rosette stage of development, while plants that we used were around 10 days younger. Using plants at different developmental stages may help to test this hypothesis.

### Changes in carbon allocation patterns within the root system

In the images taken after labelling the plants with [^18^F]FDG, it was clear that the radioactive tracer was not evenly distributed within the root system, and that the distribution changed after the experimental treatments (Figures 1 and 3). We observed a decrease in [^18^F]FDG or its metabolites to the secondary root tips in response to wounding and simulated herbivory. Root tips harbour apical meristems and are the region of both cell proliferation and cell expansion [44]. Whether the reduced carbon allocation signatures at the roots tips correlate with lower expansion and meristematic activity and contribute to root growth reduction after herbivory requires further analysis. In fact, graminaceous plants exposed to galactose in the rooting medium show similar reactions: allocation of recent photosynthates to the roots increases dramatically, but at the same time decreases into the root tips, associated with cell wall tightening and reduced elongation rate [45]. The conclusion was that solute import and growth inhibition were spatially separated within the root, which might also explain our results for *N. attenuata*. Kim et al. [46] reported decreases in disaccharide levels in sink tissues of early elongated *N. attenuata* plants within 1 h following simulated herbivory. In tomato, another Solanaceous plant, the concentrations of glucose, fructose and sucrose decreased 4 h after wounding and subsequent application of water or *M. sexta* regurgitant [19]. Future analyses of the spatial regulation of internal sugar pools in different root areas in *N. attenuata* are needed to determine how carbohydrate pools are regulated at a fine-scale in root systems.

### Regulation of allocation processes after herbivory

The nature of the signals important for the regulation of resource allocations and growth responses in roots after leaf herbivory is under debate. The oxylipin pathway, including JA and JA-Ile, is the major signalling pathway that mediates defence responses upon wounding or herbivory [47]. Simulating leaf herbivory in seedlings of *N. attenuata* also leads to the accumulation of JA in roots, and irNaCOI1 plants show somewhat higher root growth velocity than WT plants, suggesting that JA perception is, at least partially, involved in regulating this developmental response [34]. Our experiments with [^18^F]FDG also indicate that JA perception is involved in restricting wound-induced resource allocation processes (Figure 1C). However, JA is not the only plant hormone that is altered after leaf herbivory; growth-related hormones also change during herbivory (reviewed in [47]). Auxin, which is mainly supplied through the shoot apex, can be generally considered as a reporter for the integrity of apical tissues, and herbivory could strongly influence the provision of auxin from the shoot to the root system [48]. Machado et al. [32] showed transient changes in auxin levels upon leaf treatments with WOS and that external auxin applications change herbivory-induced carbohydrate and re-growth patterns. However, auxin itself is not likely to be the only messenger that induces systemic growth responses and resource allocations [49-51]. Cytokinins, whose biosynthesis and transport are inhibited by auxin [48,52-54], may play profound roles in stress-induced growth responses [55] and regulate root growth and development, such as limiting the size of the root apical meristem and the rate of root growth [56,57]. Future research will reveal how auxin, cytokinins or other hormones (e.g. abscisic acid), may change the carbon allocation and growth responses and how the JA pathway may interact with these responses.

## Conclusions

In this work we demonstrate that [^18^F]FDG is metabolized *in planta* into disaccharides and therefore provides a useful tool to study carbon allocation in plants. Using radioactive imaging techniques, we were able to detect highly localized responses at the root tip after simulated herbivory in leaves. Our results further show that JA perception is important for wound-induced carbon partitioning to leaves and roots. Future research is needed to identify if JA signalling itself or cross-talk with other hormonal sectors regulate these processes.

## Abbreviations

JA: Jasmonic acid
WW: Wounding and application of water
WOS: Wounding and application of oral secretions
WFAC: Wounding and application of fatty acid-amino acid conjugates
FDG: 2-deoxy-2-fluoro-D-glucose
